# Opioid, sedative, preadmission medication and iatrogenic withdrawal risk in UK adult critically ill patients: a point prevalence study

**DOI:** 10.1007/s11096-023-01614-9

**Published:** 2023-07-15

**Authors:** Rebekah Eadie, Cathrine A. McKenzie, Daniel Hadfield, Nicola J. Kalk, Scott Bolesta, Martin Dempster, Daniel F. McAuley, Bronagh Blackwood, UK ALERT-ICU study investigators

**Affiliations:** 1 Wellcome-Wolfson Institute for Experimental Medicine and Centre for Improving Health-Related Quality of Life, School of Psychology, Queens University Belfast, University Road, Belfast, BT7 1NN Northern Ireland, UK; 2 South-Eastern Health and Social Care Board, Trust Headquarters, Ulster Hospital, Upper Newtonards Road, Dundonald, BT16 1RH UK; 3 NIHR Biomedical Research Centre, School of Medicine, Perioperative and Critical Care Theme and NIHR Applied Research Collaborative (ARC), University of Southampton, Wessex, Southampton, S016 6YD UK; 4 Pharmacy and Critical Care, University Hospital, Southampton NHS Foundation Trust, Southampton, S016 6YD UK; 5 Centre for Human and Applied Physiological Sciences, Faculty of Life Sciences and Medicine, Florence Nightingale Faculty of Nursing, Midwifery and Palliative Care and Department of Addiction, Institute of Pharmaceutical Sciences, School of Cancer and Pharmacy, King’s College London, London, SE1 9RT UK; 6 Department of Critical Care, King’s College Hospital, London, UK; 7 Bethlam Royal Hospital, South London and Maudsley NHS Foundation Trust, Monks Orchard Road, Beckenham, BR3 3BX UK; 8 Department of Pharmacy Practice, Nesbitt School of Pharmacy, Wilkes University, 84 West South Street, Wilkes-Barre, PA 18766 USA

**Keywords:** Analgesics, Cross-sectional study, Opioids critical care, Sedatives

## Abstract

**Background:**

Iatrogenic withdrawal syndrome, after exposure medication known to cause withdrawal is recognised, yet under described in adult intensive care.

**Aim:**

To investigate, opioid, sedation, and preadmission medication practice in critically ill adults with focus on aspects associated with iatrogenic withdrawal syndrome.

**Method:**

One-day point prevalence study in UK intensive care units (ICUs). We collected ICU admission medication and/or substances with withdrawal potential, sedation policy, opioid and sedative use, dose, and duration.

**Results:**

Thirty-seven from 39 participating ICUs contributed data from 386 patients. The prevalence rate for parenteral opioid and sedative medication was 56.1% (212 patients). Twenty-three ICUs (59%) had no sedation/analgesia policy, and no ICUs screened for iatrogenic withdrawal. Patient admission medications with withdrawal-potential included antidepressants or antipsychotics (43, 20.3%) and nicotine (41, 19.3%). Of 212 patients, 202 (95.3%) received opioids, 163 (76.9%) sedatives and 153 (72.2%) both. Two hundred and two (95.3%) patients received opioids: 167 (82.7%) by continuous infusions and 90 (44.6%) patients for longer than 96-h. One hundred and sixty-three (76.9%) patients received sedatives: 157 (77.7%) by continuous infusions and 74 (45.4%) patients for longer than 96-h.

**Conclusion:**

Opioid sedative and admission medication with iatrogenic withdrawal syndrome potential prevalence rates were high, and a high proportion of ICUs had no sedative/analgesic policies. Nearly half of patients received continuous opioids and sedatives for longer than 96-h placing them at high risk of iatrogenic withdrawal. No participating unit reported using a validated tool for iatrogenic withdrawal assessment.

**Supplementary Information:**

The online version contains supplementary material available at 10.1007/s11096-023-01614-9.

## Impact statements

- In this study, a high proportion of the participating intensive care units did not have a policy for managing and weaning sedation and analgesia.
- The high prevalence of patients receiving opioid and/or sedative continuous infusion for greater than 96 h and admission medication with withdrawal potential places them at high risk of iatrogenic withdrawal.
- There is an urgent need for a validated screening tool for adult intensive care patients to assist healthcare professionals to assess for iatrogenic withdrawal syndrome.

## Introduction

Patients admitted to the intensive care unit (ICU) frequently receive opioids and sedatives for treating pain and anxiety and to facilitate effective mechanical ventilation [1]. The longer that patients receive mechanical ventilation with opioids and sedatives, the higher the risk of delirium some of which may represent iatrogenic withdrawal syndrome (IWS). IWS manifests with a combination of signs and symptoms due to dysregulation of the autonomic nervous system. These symptoms occur upon abrupt discontinuation or rapid tapering of drugs known to produce physiological dependence and the syndrome shares features of both sedative-hypnotic and opioid withdrawal. Signs of IWS overlap with delirium secondary to critical illness; and it is therefore challenging to diagnose IWS in critical illness without a validated assessment tool [2, 3].

In children, IWS is well described and associated with untoward outcomes such as an increased duration of mechanical ventilation, ICU, and hospital length of stay [3, 4]. IWS is largely unrecognised in adult intensive care and this under-recognition in adults may be due to challenges understanding the problem, its overlap with other conditions, lack of screening tools and management strategies, and its unclear impact on clinical outcome [1]. Risk factors could include prolonged and cumulative doses of opioids and benzodiazepines, prolonged duration of sedative use, high body mass index, young age, and a history of drug or alcohol dependence [5, 6].

International guidelines recommend assessment-driven, protocol-based strategies to manage pain and sedation and prevent complications, including IWS (conditional recommendation, moderate quality evidence) [1]. Within the UK, two previous sedation surveys of 214 and 157 adult ICUs respectively reported that 57 and 59% had a written sedation protocol; 94% and 78% had sedation hold policies [7, 8]. The use of IWS protocols were not reported. Furthermore, the publication of the 2018 Society of Critical Care Medicine guidelines for pain, agitation/sedation, delirium, immobility, and sleep disruption (PADIS) in adult patients in the ICU do not address IWS [1]. Clearly there are gaps in understanding of assessment, prevention, and treatment of IWS in adult ICU.

### Aim

The aim of this study w﻿as to investigate current opioid, sedation, and preadmission medication practice in critically ill adults with a focus on aspects that could relate to IWS. The study objectives were to describe how adult ICU patients were weaned from continuously administered opioids and sedatives; compare those ICUs with and without a sedation policy; describe opioids, sedatives and pre-admission medication with withdrawal potential used in participating ICUs; and identify what assessments or validated tools were used to identify IWS.

### Ethics approval

The ALERT-ICU protocol was reviewed by the Wilkes University Institutional Review Board (IRB) [Ref: #116] and was provided an ethical exempt determination notification. In the UK, the study was classified as a service evaluation, reviewed by local Research and Development Offices in participating hospitals and Data Use Agreement and Institutional Authorisation Agreements were approved.

## Method

This UK study was part of an international point prevalence study. The study was registered on ClinicalTrials.gov (AduLt iatrogEnic withdRawal sTudy in the ICU [ALERT-ICU], Bolesta 2021, NCT04422808). The study is reported in accordance with the Strengthening the Reporting of Observational Studies in Epidemiology (STROBE) Statement.

### Study design and participants

A prospective, observational, one-day point prevalence study of opioid, sedation, and drug withdrawal practices in National Health Service, NHS UK ICUs. ICUs selected a one-day study period between June 1st and September 30th, 2021. Patients aged 18-years and older admitted to adult ICUs were eligible for inclusion. Patients were included if they received either parenteral opioids and/or sedatives in the 24-h prior to the data collection day. There were no exclusion criteria.

All electronic study data were kept in password-protected computer files. Data were coded by assigning a unique identification number to participating institutions and individual ICUs and patients were assigned a unique study identification number. Analysis was performed using the coded data. Only aggregate data without personal identifiers have been included in results.

### Data collection

Recruitment of data collectors was achieved by contacting national representatives from professional networks including the UK Clinical Pharmacy Association (UKCPA), the Intensive Care Society (ICS), and the UK Critical Care Research Group (UKCCRG). Networks advertised the study on a national level and recruited members as investigators. The local investigators liaised with their Research and Development Offices to secure approval, collected data and acted as guarantor for the integrity and quality of data. To maximise consistency in reporting, registered local investigators received training on data collection through virtual meetings (led in UK by RE), and online tutorials, recorded training sessions available on the ALERT-ICU website (https://www.iatrogenicwithdrawalstudy.com/) and the Manual of Operations [9]. Anonymous patient data were collected using the Research Electronic Data Capture (Redcap) secure web-based data collection tool. The system allowed real-time input of data by local investigators.

Data were collected at the point of enrolment pertaining to ICU type, its daily interprofessional ward rounds (defined as doctor, and other disciplines including, but not limited to nurse, pharmacist, and physiotherapist) or use of opioids, sedation, admission drugs with withdrawal potential and withdrawal assessment tools and protocols. Patient characteristics and clinical data were obtained from the patient’s clinical record. Daily and cumulative amounts of opioids and sedatives were recorded along with durations of therapy and medication weaning. Patient hours on mechanical ventilation and length of ICU stay were also documented up to point of data collection.

### Medicines reconciliation data

A history of recreational substance use and long-term medication with a documented predisposal to withdrawal syndrome was also collected. The history included information about prescription of gabapentinoids, antidepressants, opioids, and history of nicotine (including tobacco), alcohol and other drug use.

### Data analysis

Data were analysed by RE, DH and BB using appropriate descriptive statistics (number, proportion; mean, standard deviation; or median, interquartile range) and are presented in tabular format. Opioid and sedative doses were expressed as total in milligrams on day of data collection. Relationships were explored between units that did/did not have sedative and opioid analgesia policies in types of analgesia and sedatives used, dosages, and reduction in dosage percentages using Chi square and Mann–Whitney U using Social Science Statistics. Risk factors associated with IWS include cumulative dose and duration of administration of 72 h or more [4, 10, 11]. Duration of opioids and/or sedative exposure was categorised into subgroups of less than 24 h, 24–72 h and greater than 96 h for analysis.

## Results

### ICU and patient characteristics

Thirty-nine ICUs from 17 UK NHS Trusts participated, and 212 of 378 screened patients (56.1%) from 37 ICUs met inclusion criteria and were included in the study (Table 1). The major ICU type was mixed medical/surgical (59%). Interprofessional bedside rounds were conducted in most (33, 84.6%), and were conducted a minimum of four times per week in 17 ICUs (43.6%). A minority of ICUs reported policies that addressed daily sedation interruption (17 ICUs, 43.6%), general sedation/analgesia (14 ICUs, 36%), sedation/analgesia weaning (8, 20.5%), and IWS 4 (10.3%). All ICUs reported having tools to assess the level of sedation only three units did not use a pain assessment tool. None of the 39 ICUs reported a validated tool to assess for IWS.

**Table 1:** ICU speciality, interprofessional ward rounds, policy, and assessment tools

	No (%)
ICUs	39
ICU patient type	
Mixed medical/surgical	23 (59.0)
Neurological	5 (12.8)
Cardiothoracic surgery	5 (12.8)
Medical	3 (7.7)
General surgical	1 (2.6)
Other	2 (5.1)
Interprofessional bedside rounds	
>/= 4 days/week	17 (43.6)
Daily	14 (35.9)
< 4 days/week	2 (5.1)
None	6 (15.4)
Sedation and analgesia policies/protocols^a^	
Daily sedation interruption	17 (43.6)
General sedation/analgesia policy, with/without daily interruption	14 (35.9)
Sedation/analgesia weaning	8 (20.5)
IWS policy to monitor signs/symptoms	4 (10.3)
Assessment tools^a^	
Sedation	39 (100)
Pain	36 (92.3)
Withdrawal	0

*IWS* Iatrogenic withdrawal syndrome

^a^Numbers do not total to 39 as some units had more than one policy/protocol or assessment tool

A greater proportion of patients were admitted with respiratory system disease (79, 37.3%) and were white (162, 76.4%) (Table 2). At the point of recruitment, patients had been in the ICU for a median of 6 (IQR 2–14) days; 165 (78.3%) were receiving invasive mechanical ventilation; and 54 (25.5%) were COVID-19 positive.

**Table 2:** Patient characteristics

*Patients (from 37 ICUs)*	212
Female	79 (37.7)
Age, median (IQR)^a^	58.0 (44.0–68.0)
BMI, kg/m^2^, median (IQR)^b^	27.3 (23.5–31.9)
Days in ICU prior to data collection, median (IQR)	6.0 (2.0–14.0)
*Ethnicity*	
Caucasian	162 (76.4)
Asian	14 (6.6)
Black/African	13 (6.1)
Other	3 (1.4)
Unknown	20 (9.4)
*COVID-19 status*	
PCR positive	54 (25.5)
*Reason for ICU admission*	
Respiratory system disease	79 (37.3)
Circulatory system disease	37 (17.5)
Nervous system disease	28 (13.2)
Digestive system disease	27 (12.7)
Other	41 (19.3)
*Preadmission medications*	
Antidepressants/antipsychotics	43 (20.3)
Opioids	30 (14.2)
Paracetamol	22 (10.4)
Gabapentin or Pregabalin	18 (8.5)
Non/benzodiazepine sleeping medication	10 (4.7)
NSAIDs	7 (3.3)
*Preadmission nicotine, alcohol, and recreational drug use*	
Nicotine	41 (19.3)
Alcohol misuse	25 (11.8)
Recreational/illicit drugs	12 (5.7)
*Mechanical ventilation treatment in the ICU*	
Invasive mechanical ventilation	165 (77.8)
Non-invasive ventilation	8 (3.8)
*Parenteral opioid and sedative use over previous 24 h (inclusion criteria)*	
Opioid	202 (95.3)
Sedative	163 (76.9)
Received BOTH opioid and sedative	153 (72.2)
Received ONLY opioid	49 (23.1)
Received ONLY sedative	10 (4.7)

Patients were recruited from 37 out of the 39 ICUs

Data are number (%) of patients unless otherwise stated

*IWS* Iatrogenic withdrawal syndrome, *IQR* Interquartile range, *BMI* Body mass index, *PCR* Polymerase chain reaction

^a^n = 191; missing data for 21 patients

^b^n = 178; missing/unknown = 34

The main medications with iatrogenic withdrawal potential taken by patients prior to admission were antidepressants or antipsychotics (20.3%) and opioids (14.2%), with 41 (19.3%) taking nicotine and 12 (5.7%) with a history of taking recreational drugs (Table 2). Alcohol misuse was noted in 25 (11.8%) patients.

In the 24-h prior to data collection, 202 patients (95.3%) received parenteral opioids; 163 (76.9%) received parenteral sedatives; and 153 (72.2%) received both.

### Opioids

Of 202 patients who received parenteral opioids the most used were fentanyl (35.1%) and alfentanil (33.2%) (Table 3). There were 167 (83%) patients who received opioids by continuous infusion and 44.6% received opioids for more than 96 h. 150 patients received opioids for 24 h or more, and 36.3% had the dose reduced within the previous 24 h. Sixteen patients (29.6%) had their dose reduced by more than 50%. 171 (84%) patients were receiving the shorter acting opioids, remifentanil, alfentanil and fentanyl.

**Table 3:** Opioid use over 24 h

	All	ICU with sedation policy	ICU without sedation policy
*Patients receiving opioids, N (%)*^a^	*202*	*78*	*124*
Fentanyl	71 (35.2)	49 (62.8)	22 (17.7)
Alfentanil	67 (33.2)	15 (19.2)	52 (41.9)
Remifentanil	33 (16.3)	13 (16.7)	20 (16.1)
Oxycodone^b^	20 (9.9)	0	20 (16.1)
Morphine	15 (7.4)	2 (2.6)	13 (10.5)
Tramadol^b^	2 (1.0)	0	2 (1.6)
Methadone^b^	1 (0.5)	0	1 (0.8)
*Methods of administration, N (%) of patients receiving opioids *via* each route*^a, c^			
Continuous IV infusions	167 (82.7)	68 (87.2)	99 (79.8)
PCA	21 (10.4)	8 (10.3)	13 (10.5)
Non-scheduled intermittent	11 (5.4)	0 (0)	11 (8.9)
Scheduled intermittent	7 (3.5)	1 (1.3)	6 (4.8)
Regional anesthesia	2 (1.0)	1 (1.3)	1 (0.8)
*24-h dose *via* continuous IV infusion, mg, Median (IQR)*			
Fentanyl	2.9 (1.4–4.8)	3.5 (1.7–5.5))	1.9 (0.8–3.9)
Alfentanil	48.0 (32.5–92.0)	96.0 (38–120)	43.0 (25.3–65.9)
Remifentanil	16.1 (4.8–21.7)	14.6 (5.3–24.8)	16.1 (4.4–18.5)
Oxycodone	50.5 (40.0–61.0)	0 (0)	50.5 (40.0–61.0)
Morphine	208.0 (66.0–240.0)	344.0 (208.0–480.0)	144.0 (5.0–240.0)
*Duration of opioid treatment to the point of data collection*			
< 24 h	52 (25.7)	23 (29.5)	29 (23.4)
24–72 h	47 (23.3)	15 (19.2)	32 (25.8)
72–96 h	13 (6.4)	4 (5.1)	9 (7.3)
> 96 h	90 (44.6)	36 (46.2)	54 (43.5)
*Opioid reduction in previous 24 h*^*e*^* N (%)*			
Yes	54 (36.0)	23 (41.8)	31 (32.6)
*Reduction % in previous 24 h*^*e*^* N (%)*			
< 10%	3 (5.6)	1 (4.3)	2 (6.5)
10–20%	12 (22.2)	7 (30.4)	5 (16.1)
21–30%	12 (22.2)	5 (21.7)	7 (22.6)
31–50%	11 (20.4)	2 (8.7)	9 (29.0)
> 50%	16 (29.6)	8 (34.8)	8 (25.8)
*Enteral opioids started in previous 24 h*^*e*^* N (%)*			
Yes	11 (5.4)	7 (63.6)	4 (36.4)

Data are number (%) of patients, unless otherwise stated

^a^Numbers different to column total as some patients received more than one type of opioid

^b^These opioids were free form entries

^c^CA = Patient Controlled Analgesia (Non-scheduled intermittent refers to one-off or as needed intravenous, subcutaneous, or intramuscular doses; Scheduled intermittent refers to single, non-continuous intravenous, subcutaneous, or intramuscular doses administered according to a schedule

^d^Fentanyl equivalent conversion

^e^Only patients receiving opioids for 24 h or more

Within 14 ICUs that had a sedation/analgesia policy in place there were more patients on fentanyl and less patients on alfentanil and morphine (*X*^*2*^ 36.87 [df 3], *N* = 186, *p* = < 0.001). No patients in ICUs with a policy received oxycodone in comparison to 20 patients in non-policy ICUs. For patients with an opioid duration of 24 h or more; there was no relationship between policy/no policy ICUs and opioid duration (*X*^*2*^ 1.99 [df 3], *N* = 202, *p* = 0.57) or the proportionate reduction in opioids over the previous 24-h (*X*^*2*^ 4.37 [df 3], *N* = 54, *p* = 0.36).

Higher doses of fentanyl as continuous infusion were administered to patients in ICUs with no sedation/analgesia policy in comparison to those with a policy (Mann–Whitney test (two-tailed) N = 71, *p* = 0.047. In contrast, doses of alfentanil doses were higher in ICUs with a sedation/analgesia policy and these ICUs recorded higher total overall opioid exposure (Mann–Whitney test (two-tailed) alfentanil, N = 67 *p* = 0.0029.

### Sedatives

One hundred and sixty-three patients received sedatives and the most common was propofol (83.4%), followed by midazolam (20.2%) (Table 4). The main method of administration was continuous infusion, and 45.4% of patients received sedatives for more than 96 h. 120 patients received sedatives for 24 h or more, and 36.7% patients had a reduction in dosage and 31.8% had their dose reduced by more than 50%.

**Table 4:** Sedative use over 24 h

Variable	All	ICU with sedation policy	ICU without sedation policy
*Patients receiving sedatives, N (%)*^a^	163	64	99
Propofol	136 (83.4)	57 (89.1)	79 (79.8)
Midazolam	33 (20.2)	12 (18.8)	21 (21.2)
Clonidine	17 (10.4)	5 (7.8)	12 (12.1)
Dexmedetomidine	9 (5.5)	3 (4.7)	6 (6.1)
Ketamine	2 (1.2)	1 (1.6)	1 (1)
Lorazepam	3 (1.8)	0 (0)	3 (3)
*Methods of administration, N (%) of patients receiving sedatives via each route*^a, b^			
Continuous infusion	157 (77.7)	63 (98.4)	94 (94.9)
Scheduled intermittent	7 (3.5)	2 (3.1)	5 (5.1)
Non-scheduled intermittent	5 (2.5)	1 (1.6)	4 (4.0)
*Cumulative 24-h dose via continuous IV infusion, (mg), median (IQR)*			
Propofol	2680.0 (1000.0–4520.0)	2840.0 (960.0–4697.5)	2600.0 (1050.0–4520.0)
Midazolam	158.0 (99.4–240.0)	191.5 (95.8–240.0)	144.0 (99.4–238.0)
Clonidine	1.1 (0.4–2.5)	0.4 (0.3–2.2)	1.3 (0.9–2.9)
Dexmedetomidine	2.0 (0.9–2.3)	2.0 (0.7–2.4)	1.7 (0.9–2.5)
Ketamine	230.0 (230.0–230.0)	230.0 (230.0–230.0)	–
*Duration of sedative treatment to the point of data collection*			
< 24 h	43 (26.4)	16 (25.0)	27 (27.3)
24–72 h	36 (22.1)	12 (18.8)	24 (24.2)
72–96 h	10 (6.1)	5 (7.8)	5 (5.0)
> 96 h	74 (45.4)	31 (48.4)	43 (43.4)
*Sedative reduction in previous 24 h*^c^			
Yes	44 (36.7)	19 (29.7)	25 (25.3)
*Reduction % in previous 24 h*^c^			
< 10%	3 (6.8)	0 (0)	3 (12.0)
10–20%	10 (22.7)	6 (31.6)	4 (16.0)
21–30%	7 (15.9)	2 (10.5)	5 (20.0)
31–50%	10 (22.7)	5 (26.3)	5 (20.0)
> 50%	14 (31.8)	6 (31.6)	8 (32.0)
*Enteral sedative started in previous 24 h*^c^			
Yes	11 (9.2)	4 (21.1)	7(28.0)

Data are number (%) of patients, unless otherwise stated

^a^Numbers different to column total as some patients received more than one type of sedative

^b^PCA = Patient Controlled Analgesia (Non-scheduled intermittent refers to one-off or as needed intravenous, subcutaneous, or intramuscular doses; Scheduled intermittent refers to single, non-continuous intravenous, subcutaneous, or intramuscular doses administered according to a schedule

^c^Only patients receiving opioids for 24 h or more

There was no significant relationship between policy/no policy ICUs and types of sedative used (X^2^ 1.42 [df 4], N = 197, *p* = 0.84), duration of sedative use (X^2^ 1.3 [df 3], N = 163, *p* = 0.73), or the proportionate reduction over the previous 24-h period (X^2^ 1.7 [df 3], N = 41, *p* = 0.62). For those patients receiving sedatives for greater than 72 h, there was a later increase in alpha-2-agonists and midazolam use. (Table 5 in supplementary file 1).

### Medicines reconciliation data

From a total of 212 patients, there were 178 pre-ICU admission historical prescriptions for medications associated with a withdrawal syndrome, or a medical history of alcohol, nicotine, or substance dependence.The overall prescription rate was 0.47 per included patient. The highest rate was for antidepressants/antipsychotics at 20.3%, followed by nicotine dependence (including tobacco) in 19.3% of patients, followed by long term opioids (14.2%), and 11.8% had a report of alcohol abuse.

## Discussion

This prospective, observational, one-day point prevalence study reported: (1) high exposure of ICU patients to continuous infusions of opioids and sedatives, with over 50% of participants receiving continuous sedation or opioids for more than 72 h; (2) a higher incidence of opioid than sedative administration; (3) heterogenous practice relating to sedation and opioid use, including medication choice, sedation policy and weaning strategies; (4) an absence of validated tools to allow identification and treatment of IWS; (5) limited use of policies or protocols to guide sedation and opioid practice; and (6) high prevalence of preadmission substances and medication known to cause a withdrawal syndrome.

This study gives contextual information for IWS in the adult ICU population; and gives evidence for IWS risk in adults. The majority of IWS research literature has been derived from work conducted in the paediatric critical care population where IWS is recognised, assessed with validated tools (benzodiazepine and opioid withdrawal scale (SOPHIA); clinical opioid withdrawal scale (COWS)) and managed with longer acting opioid agents including methadone [4, 6]. In the paediatric critical care literature, exposure to opioids or sedatives for greater than 72 h is deemed higher risk for IWS. In the context of this point prevalence, almost half of included patients could be at risk of developing IWS. There are no validated IWS tools for adult ICU patients, although there are validated tools for adults to screen for alcohol and substance withdrawal, but these are not suitable for the ICU setting. Finally, the study was conducted in 39 UK ICUs and therefore gives a broad perspective of IWS risk and include prevalence preadmission medication with withdrawal potential opioid and sedative exposure.

There were limitations to the point prevalence data .78 ICUs originally invited to participate with only 39 (50%) ICUs finally contributing their data. One in four of ICU patient admissions were admitted with Covid-19 related pathophysiology and thus the data may not reflect ICU admissions during a non-pandemic time. Relevant clinical outcome data after the day of point prevalence, including duration of mechanical ventilation of ICU length of stay, were not collected. Alcohol and nicotine dependence is widely acknowledged to be underreported. Furthermore, the data were derived using observational point prevalence methodology and were dependent on patient demographic and opioid/sedation data on the day of data collections, thus there may be a risk of selection bias.

The proportion of opioid administration was high in patients in comparison to sedatives. Whether or not this is a consequence of recent guidelines published by the Society of Critical Care Medicine (SCCM 2018) that recommend that pain is treated before considering sedation is difficult to establish [1]. An assessment driven, protocol-based approach to pain and sedation management is recommended in PADIS [1]. Such an approach was not evident in the findings that reported less than half of ICUs had interprofessional rounds, just over a third had general policies for sedation and analgesia, and very few ICUs with guidelines for weaning medications and monitoring for signs of IWS. The lack of monitoring is contrary to the general view that during the reduction of sedative-analgesic medications, patients should be closely monitored for acute withdrawal phenomenon Indeed, no ICUs used any validated withdrawal screening tool and there is currently no validated IWS screening tool for adult ICU.

In this dataset, five different opioids were administered mainly by continuous infusion (alfentanil, fentanyl, morphine, remifentanil, and oxycodone). All five have different duration of action and affinity for the μ receptor. Whether different opioids increase the risk of IWS is yet to be fully established. Although there are reports in the literature that the ultra-short acting opioid, remifentanil with a very high affinity for the μ receptor increase the risk of IWS and hyperalgesia [12]. Finally more fentanyl and less of the shorter acting alfentanil was used in ICUs with a sedation policy, whether the shorter duration of alfentanil means more IWS is not known [12].

Accepting the bias of observation data collected using single day point prevalence methodology, we suggest these findings give evidence of high risk of IWS in adult ICU patients, in patients receiving opioids and sedatives by continuous infusion for greater than 72 h and preadmission medication with a high risk of withdrawal.

Close to half (44.6%) of patients receiving opioids had a continuous infusion for 96 h or more, with a dose reduction in the previous 24 h in only 3 (5.6%) patients. Thus, if most patients were on short acting agents (n = 171 (84%) for 96 h or greater; this might have a greater impact on IWS risk. In 2021, Maffei et al. assessed risk factors for IWS in an adult Covid-19 ICU population. Using a multivariable model they showed that each additional day of IV opioid therapy was associated with an 8% increase in odds of IWS (95% CI 1.02–1.14) [3]. However, their findings should be treated with caution, as they did not use a validated assessment tool. Instead, they defined and measured IWS as the receipt of scheduled oral opioid, benzodiazepine, and/or clonidine regimens after cessation of IV analgesics and sedatives while in the ICU. They concluded prolonged and high dose exposures to IV opioids and benzodiazepines should be limited when feasible. Further, Arroyo et al. reported that in 50 ICU patients receiving benzodiazepines and/or opioids, of which 84% of patients were taking a mixture of midazolam (84%) and lorazepam (70%), probable withdrawal syndrome occurred in 55% of patients.

With respect to sedatives, propofol was the most used agent in (n = 136 (85%) of patients which corresponds with international sedative guidance (e.g., PADIS 2018) [1]. What was perhaps surprising was that one in five patients were receiving midazolam despite international guidelines that advise against benzodiazepines, especially midazolam and lorazepam, because of risk of delirium and over sedation [13, 14]. The higher use of benzodiazepines may have reflected the high prevalence (25.2%) of ICU Covid-19 admissions during data collection. Greater use of benzodiazepines and more challenging sedation were reported in Covid-19 ICU admissions by Pun et al. in 2020 and Hanks et al. in 2022 [15, 16]. Maffei et al. reported a higher incidence of IWS after benzodiazepine administration, the risk being 3 times higher after receiving lorazepam (95% CI 1.12–8.15).

Potential risk factors for IWS were present in 47% of patients pre-admission, these were alcohol or nicotine dependence or presence of chronic medication that have withdrawal symptoms on cessation including gabapentinoids and antidepressants. We speculate that most patients would have had these medications withheld on ICU admission (especially if the oral or enteral route is not available) and this could contribute to IWS.

Future research should include: (1) Development and validation of tools for IWS detection in adult ICU patients (2) Establish whether opioid dose, choice and duration impacts the risk of IWS in the adult ICU population (3) Ascertain if greater use of alpha-2-agonists over propofol and benzodiazepines, known to manage opioid, alcohol and nicotine, reduce likelihood IWS [17, 18].

## Conclusion

This prospective, observational, one-day point prevalence study conducted in 39 National Health Service UK ICUs showed a high incidence of opioid and sedation administration, with almost half of ICU admissions receiving opioids for over 96 h. There was high prevalence of preadmission medication and substances with withdrawal potential thereby likely increasing the risk of IWS in adult ICU.

### Supplementary Information

Below is the link to the electronic supplementary material.

Supplementary file1 (DOCX 15 KB)

Supplementary file2 (DOCX 21 KB)
