# Supplementary material for: Opioid, sedative, preadmission medication and iatrogenic withdrawal risk in UK adult critically ill patients: a point prevalence study
Source: Int J Clin Pharm. 2023 Jul 15;45(5):1167–75. doi: 10.1007/s11096-023-01614-9 (PMC10600273; doi:10.1007/s11096-023-01614-9)
Supplement: Supplementary file 1 — Supplementary file1 (DOCX 15 KB) [file 11096_2023_1614_MOESM1_ESM.docx]

Table 5. Relationship between sedative use and time

| Continuous sedative duration treatment | <24 hours  N=43 | 24  < 72 hours   N= 36 | 72 < 96 hours  N=10 | 96 hours  N=74 |
| --- | --- | --- | --- | --- |
| Propofol | 38 (88.4) | 28 (71.8) | 9 (90.0) | 61 (82.4) |
| Dexmedetomidine | 1 (2.3) | 2 (5.6) | 1 (10.0) | 5 (6.8) |
| Ketamine | 1 (2.3) | 1 (2.8) | 0 | 0 |
| Lorazepam | 1 (2.3) | 1 (2.8) | 0 | 1 (1.4) |
| Midazolam | 5 (11.6) | 8 (20.5) | 0 | 20 (27.0) |
| Clonidine | 2 (4.7) | 2 (5.6) | 0 | 13 (17.6) |
