# Supplementary material for: Opioid, sedative, preadmission medication and iatrogenic withdrawal risk in UK adult critically ill patients: a point prevalence study
Source: Int J Clin Pharm. 2023 Jul 15;45(5):1167–75. doi: 10.1007/s11096-023-01614-9 (PMC10600273; doi:10.1007/s11096-023-01614-9)
Supplement: Supplementary file 2 — Supplementary file2 (DOCX 21 KB) [file 11096_2023_1614_MOESM2_ESM.docx]

**Supplementary File 2.**

**List of ALERT-ICU Investigators**

List of ALERT-ICU collaborators: site principal investigators - Belinda Badman, Princess Alexandra Hospital, Harlow, United Kingdom; Sanchia Barnes, Manchester University NHS Foundation Trust, Manchester, United Kingdom; Shady Botros Ninewells Hospital, NHS Tayside, Dundee, Scotland; Odran Farrell, University Hospital Plymouth NHSFT, Plymouth, United Kingdom; Pietro Ferranti, Lister Hospital, Stevenage, East & North Herts NHS Trust, Stevenage United Kingdom; Emma Graham-Clarke, Sandwell and West Birmingham NHS Trust, West Bromwich, United Kingdom; Fraser Hanks, Guy's and St Thomas' NHS Foundation Trust, London, United Kingdom; Joanne Hanley, Antrim Area Hospital, Antrim, Northern Ireland; Jaspreet Kaur, The Royal Wolverhampton NHS Trust, Wolverhampton, United Kingdom; Claire Kohler, Royal Berkshire, Reading, United Kingdom; Kate Leonardo, Wye Valley NHS Trust, Hereford, United Kingdom; Anna Man, Mid & South Essex NHS Foundation Trust, Chelmsford, United Kingdom; Iain McCullagh, The Newcastle Hospital NHS, Newcastle, United Kingdom; Jill McDonald, Milton Keynes University Hospital NHS Foundation Trust, Milton Keynes, United Kingdom; Judith Niland, Leeds Teaching Hospitals NHS Trust, Leeds, United Kingdom; Madeline O'Hare, The Royal Wolverhampton NHS Trust, Wolverhampton, United Kingdom; Christopher Remmington, Guy's and St Thomas' NHS Foundation Trust, London, United Kingdom; Jas Sall, Sheffield Teaching Hospitals NHS Foundation Trust, Sheffield, United Kingdom; Jonathan Smith, Royal Berkshire, Reading, United Kingdom; Alan Timmins, Victoria Hospital, NHS Fife, Scotland; Thu Ha Trinh, St George's University Hospitals NHS Foundation Trust, London, United Kingdom; Sinead Tynan, Barking, Havering and Redbridge University Hospitals Trust, Havering, United Kingdom; Franki Wilson, Leeds Teaching Hospitals NHS Trust, Leeds, United Kingdom.

All data collectors where either registered with the General Pharmaceutical Council, Pharmaceutical Society of Northern Ireland, General Medical Council or The Nursing and Midwifery Council.
